# Recent progress in cadonilimab research for oncology applications

**DOI:** 10.3389/fimmu.2025.1694490

**Published:** 2026-01-14

**Authors:** Ming-Zhen Dong, Ming Cui, En-Bo Zhu, Ming-Quan Lin, Guang-Hui Dong, Guang-Lin Jin, Lin-Zhuo Qu, Hui-Ying Che, Hong-Jian Guan

**Affiliations:** 1 Department of Neurology, Yanbian University Hospital, Yanji, Jilin, China; 2 Reproductive Medicine Center, Yanbian University Hospital, Yanji, Jilin, China; 3 Department of General Medicine, Yanbian University Hospital, Yanji, Jilin, China

**Keywords:** bispecific antibody, cadonilimab, immunotherapy, lung cancer, other malignancies

## Abstract

Cadonilimab is the first bispecific antibody independently developed in China that simultaneously targets Programmed Cell Death Protein-1 (PD-1) and Cytotoxic T Lymphocyte-Associated Antigen-4 (CTLA-4), marking a significant milestone in both clinical applications and drug development. Through its dual mechanism of action, cadonilimab blocks PD-1 and CTLA-4 signaling pathways concurrently, thereby activating T cells and enhancing antitumor immune responses. Within the tumor microenvironment, cadonilimab promotes effector T-cell infiltration while reducing nonspecific attacks on normal tissues, thus lowering the incidence of immune-related adverse events. In comparison to conventional monospecific antibodies, cadonilimab exhibits superior selectivity and safety. Multiple studies have shown that, either as monotherapy or in combination regimens, cadonilimab exhibits promising antitumor activity and tolerability in refractory solid tumors such as advanced cervical cancer, hepatocellular carcinoma, non-small cell lung cancer, and gastric cancer, with notable efficacy even in patients with low or negative PD-L1 expression. The successful development of cadonilimab not only underscores China’s innovative capabilities in the field of cancer immunotherapy but also provides valuable insights for global drug development and clinical practice. However, most signals derive from phase I/II single-arm or small-sample studies with limited follow-up, and no randomized head-to-head trials have yet confirmed superiority over standard PD-1+CTLA-4 approaches. This review summarizes the mechanism of action, structural characteristics, clinical research progress, and future applications of cadonilimab, with the aim of offering a useful reference for research and clinical treatment while promoting its broader application in oncology.

## 1 Introduction

### 1.1 Background and rationale

Cadonilimab is the first bispecific antibody independently developed in China and the world’s first dual-target antibody against Programmed Cell Death Protein-1 (PD-1) and Cytotoxic T Lymphocyte-Associated Antigen-4 (CTLA-4) to enter clinical trials. Although results are promising, its current approval is limited to advanced cervical cancer, with broader benefits still requiring further investigation. At present, this agent is primarily approved for the treatment of advanced cervical cancer and has demonstrated promising antitumor activity across multiple refractory malignancies (1). This review will focus on the mechanism of action, structural characteristics, and the latest clinical research progress of cadonilimab in oncology, with the aim of providing theoretical insights and practical reference for its broader application in clinical practice.

### 1.2 Cadonilimab as a PD-1/CTLA-4 bispecific

Cadonilimab represents the latest advancement in immune checkpoint inhibitors (ICIs) and is designed to address limitations of single-pathway blockade. ICIs enhance T-cell activity by blocking PD-1/PD-L1, CTLA-4 and other checkpoints, but single-pathway inhibition yields modest response rates and multi-ICI combinations often raise immune-related adverse events (2–11).

With continuous advances in antibody engineering, researchers have developed BsAbs featuring two binding sites. These antibodies are capable of simultaneously recognizing two distinct antigens or two different epitopes on the same antigen, thereby enabling highly efficient immune modulation (12, 13). BsAbs vary in size, half-life, flexibility, and tissue penetration (14). Based on their checkpoint specificity, BsAbs can be broadly categorized into those targeting dual inhibitory checkpoints, co-stimulatory and inhibitory checkpoints, or checkpoint and non-checkpoint immunomodulatory targets (15). Clinical studies have shown that, compared with combination therapy, BsAbs exhibit stronger antitumor activity while maintaining irAEs within a manageable range (15). Moreover, by fine-tuning the affinity of their dual binding sites, BsAbs can minimize off-target effects, offering new hope for cancer treatment. Beyond PD-1/CTLA-4, next-generation bispecifics target alternative checkpoints. Tebotelimab (MGD013), a PD-1×LAG-3 DART, showed an ORR ~13% across advanced solid tumors with grade ≥3 immune-related AEs ~14%, while EBV-associated lymphomas demonstrated higher response rates (16). XmAb841 (a PD-1×LAG-3 IgG1 bispecific) produced an ORR ~15% in a phase I study of solid tumors with manageable safety (grade ≥3 TRAEs ~12%) (17). Sabestomig (RO7247669/RG7769), a PD-1×TIM-3 bispecific, reported ORR 10–20% in early NSCLC/HNSCC cohorts with grade ≥3 TRAEs ~16–20% (18). These data indicate that dual targeting of emerging checkpoints can deliver antitumor activity with tolerable safety, providing a benchmark to contextualize cadonilimab’s PD-1/CTLA-4 approach.

**Figure 1:**
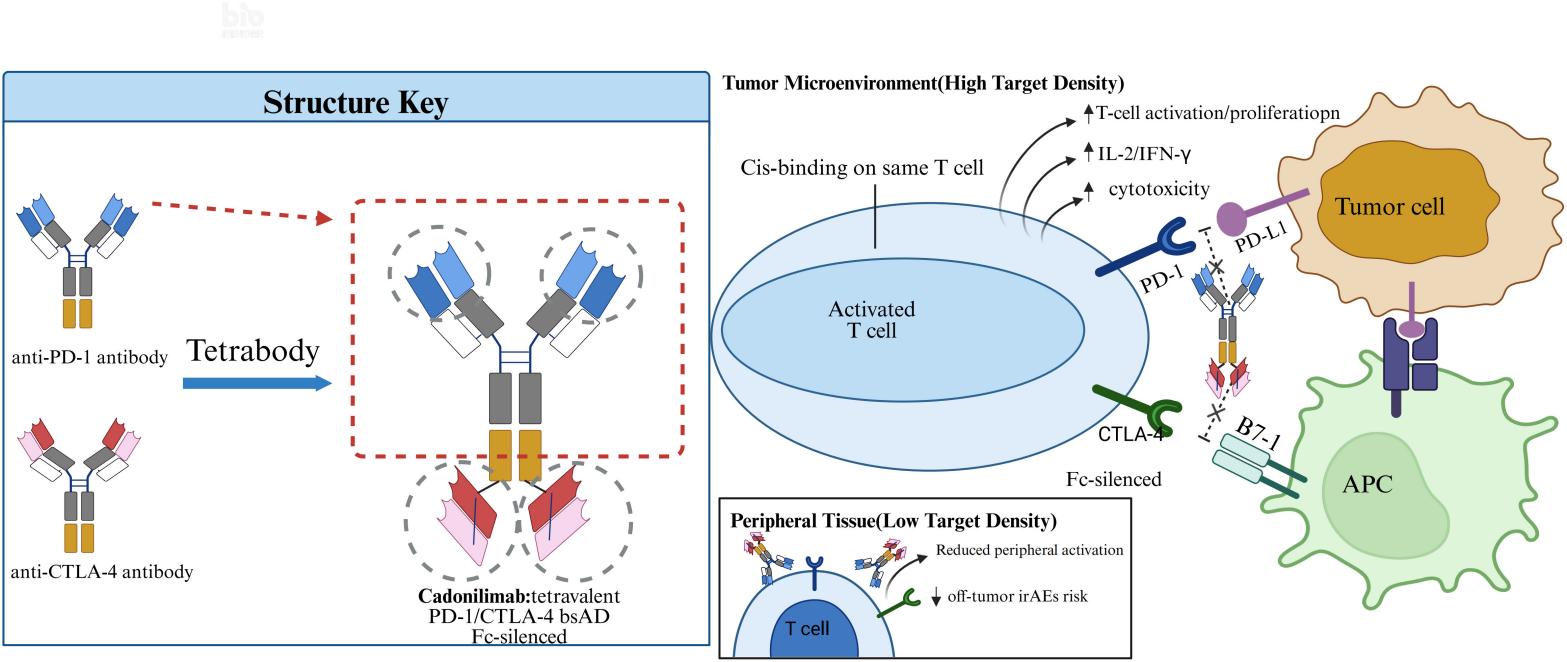
Cadonilimab is a tetravalent PD-1/CTLA-4 bispecific antibody designed to simultaneously bind PD-1 and CTLA-4 on the same T cell in high-density tumor microenvironments. It enhances T-cell activation and cytotoxicity by blocking PD-1/PD-L1 and CTLA-4/B7 signaling. Fc-silencing prevents ADCC/CDC, and reduced peripheral activation minimizes the risk of immune-related adverse events.

Cadonilimab simultaneously targets PD-1 and CTLA-4, blocking the interaction of PD-1 with its ligands PD-L1/PD-L2 and of CTLA-4 with its ligands B7-1/B7-2, thereby exhibiting comparable competitive binding activity (**Figure 1**) (19–21). PD-1 mainly dampens effector T-cell function within the tumor bed, whereas CTLA-4 curtails early T-cell priming and Treg-mediated suppression; dual blockade yields synergistic activation while limiting compensatory escape (19, 20, 22, 23). Antibody engineering of cadonilimab is a tetravalent symmetric IgG1-like design carries two PD-1 and two CTLA-4 arms with Fc silencing to minimize ADCC/CDC, and cis binding boosts avidity when PD-1 and CTLA-4 co-occur on the same T cell, strengthening local checkpoint blockade, the bispecific can engage PD-1 and CTLA-4 on one T cell or across a T cell–APC synapse, promoting receptor clustering; reduced FcγR engagement lowers peripheral crosslinking and off-tumor activation risk (19, 24). Cadonilimab demonstrates preferential and high-affinity binding to PD-1 and CTLA-4 within tumor microenvironments with elevated expression of these targets. This binding is accompanied by reduced Fc effector function, which is anticipated to result in fewer systemic immune-related adverse events compared to the combination therapy of PD-1 and CTLA-4 monoclonal antibodies (19, 20, 24). By inhibiting both PD-1 and CTLA-4 signaling pathways, cadonilimab promotes T-cell activation and proliferation, accompanied by increased secretion of cytokines such as interleukin-2 (IL-2) and interferon-γ (IFN-γ). This restores the cytotoxic function of T cells against tumor cells and enhances their antitumor capacity (24).

Cadonilimab exhibits higher affinity for tumor-infiltrating lymphocytes than for peripheral lymphocytes, thereby promoting the enrichment of effector T cells within the tumor microenvironment while reducing the activation and distribution of peripheral T cells (25). This mechanism enables activated T cells to concentrate their cytotoxic activity on tumor cells, minimizing nonspecific damage to normal tissues and lowering the risk of off-tumor toxicities (26). In addition, its unique tetravalent symmetric structure and Fc design facilitate accumulation in tumor tissues, further enhancing antitumor efficacy. Cadonilimab also demonstrates stronger binding affinity in environments with high densities of PD-1 and CTLA-4, achieving potent clinical effects with reduced toxicity (27). Compared with conventional PD-1 plus CTLA-4 combination therapy, cadonilimab shows significantly lower toxicity while providing superior safety and efficacy (19). Although it has only recently been approved, cadonilimab has already demonstrated broad therapeutic potential and promising clinical prospects in China. Nevertheless, published data are mostly early-phase without randomized controls, and the long-term safety/efficacy profile requires confirmation in larger comparative trials.

Beyond cadonilimab, multiple PD-1/CTLA-4 bispecific antibodies are in development. Vudalimab (XmAb20717) uses an IgG1 heterodimer format; a phase I trial across solid tumors reported an ORR around 14% with grade ≥3 immune-related AEs ~30%, with higher signals in endometrial and prostate cohorts (28). Volrustomig (MEDI5752) employs asymmetric monovalent binding that biases CTLA-4 blockade toward PD-1–positive T cells; early renal cell carcinoma data showed ORR 23–34% but grade ≥3 treatment-related AEs of 43–58% (23, 29). MGD019, a PD-1–guided CTLA-4 DART, produced an ORR of 13% and grade ≥3 immune-related AEs of 18% in heavily pretreated solid tumors (22, 30). Cadonilimab’s tetravalent, tumor-enriched binding aims to retain dual-checkpoint efficacy while reducing peripheral toxicity compared with these agents and historical PD-1+CTLA-4 combinations (19, 20).

Cadonilimab’s dual PD-1/CTLA-4 targeting is intended to deliver synergistic T-cell activation and reduce compensatory escape compared with single ICIs (19, 20, 22, 23). Its tetravalent symmetric architecture (two PD-1 and two CTLA-4 arms) with Fc silencing minimizes ADCC/CDC, while cis-binding increases avidity when PD-1 and CTLA-4 co-exist on the same T cell, enriching blockade in the tumor microenvironment and potentially lowering peripheral irAEs versus two-monoclonal combinations (19–21, 24). Early trials report manageable grade ≥3 irAEs (~20–30%) alongside antitumor activity, consistent with this engineered safety rationale (20, 27).

## 2 Therapeutic efficacy and safety profile of cadonilimab across malignancies

### 2.1 Cadonilimab in cervical cancer: research progress and clinical application

Cadonilimab has demonstrated broad applicability in the treatment of patients with recurrent or metastatic (R/M) cervical cancer, showing favorable efficacy and manageable safety. Evidence suggests activity across PD-L1 strata, but subgroup data are exploratory: in one phase II trial ORR was 43.8% in PD-L1–positive versus 16.7% in PD-L1–negative tumors, indicating a numerical difference and the need for larger, powered analyses before concluding PD-L1–independence (31). In a phase II clinical trial involving previously treated patients with advanced cervical cancer, the objective response rate (ORR) at 6 and 12 months was 33.0% and 12.0%, respectively, while the duration of response (DOR) at 6 and 12 months reached 77.6% and 52.9%, respectively. Subgroup analysis further revealed that the ORR was 43.8% among PD-L1–positive patients and 16.7% among PD-L1–negative patients. Treatment-related adverse events (TRAEs) of grade ≥3 occurred in 28.8% of patients, with anemia and decreased appetite being the most commonly reported (31). Grade ≥3 TRAEs of 28.8% appear higher than pembrolizumab monotherapy in R/M cervical cancer (~12–15% in KEYNOTE-158) yet lower than historical PD-1+CTLA-4 combinations (~30–40% in CheckMate 358); cross-trial comparisons remain hypothesis-generating only (32, 33).

In a phase 1b/2 trial, cervical cancer patients treated with cadonilimab (6 mg·kg^−1^, Q2W) achieved an ORR of 32.3%. Among patients with a PD-L1 positivity score ≥1, the ORR was 42.9%. The incidence of grade ≥3 TRAEs was 28%, while grade ≥3 irAEs occurred in 14% of patients. Notably, PD-L1–positive patients with recurrent or metastatic (R/M) cervical cancer also demonstrated radiographic remission following cadonilimab therapy, with the response maintained for up to 10 months (34).

Updated 2024 phase 2 COMPASSION-13 data (first-line cadonilimab + chemotherapy ± bevacizumab) reported ORR 66.7–92.3% across dosing cohorts with grade ≥3 irAEs ~20–27%, reinforcing feasibility as a frontline option (35). In cohort A-15, the highest incidence of irAEs was observed, with grade ≥3 irAEs occurring in 26.7% of patients. These findings suggest that higher dosing of cadonilimab may be positively associated with an increased risk of adverse events.

In addition, a 59-year-old patient with advanced cervical cancer, who was unable to continue chemotherapy due to grade IV chemotherapy-induced myelosuppression, received cadonilimab treatment after exclusion of contraindications. Upon evaluation, the patient achieved a complete response (CR), which was sustained through the most recent follow-up. Cadonilimab therapy markedly improved the patient’s quality of life (36). This case provides new clinical insights, suggesting that cadonilimab may serve as a therapeutic option for advanced cervical cancer patients who experience failure of, or intolerance to, standard chemotherapy. This single complete response after chemotherapy intolerance is illustrative and anecdotal, representing very low-level evidence not generalizable.

Cadonilimab has now been approved by the National Medical Products Administration (NMPA) for the treatment of advanced cervical cancer in China, achieving favorable clinical outcomes. At present, three phase II clinical trials (ChiCTR2400085618, ChiCTR2400083624, ChiCTR2300076740) are being conducted domestically, which are expected to provide more robust clinical evidence to further support its application in clinical practice.

### 2.2 Cadonilimab in hepatocellular carcinoma: research progress and clinical application

Beyond cervical cancer, cadonilimab has also shown emerging therapeutic potential in hepatocellular carcinoma (HCC). In 2023, Qiao et al. conducted a phase Ib/II clinical trial to evaluate the efficacy of cadonilimab in combination with lenvatinib in patients with advanced HCC. The results demonstrated that, although no patients achieved a CR, 21 patients (35.6%) experienced a partial response (PR), with 11 cases in cohort A (cadonilimab 6 mg·kg^−1^, Q2W + lenvatinib) and 10 cases in cohort B (cadonilimab 15 mg·kg^−1^, Q3W + lenvatinib). The disease control rate (DCR) and objective response rate (ORR) were 90.3% and 35.5% in cohort A, and 92.9% and 35.7% in cohort B, respectively. In a first-line Ib/II single-arm study (primarily including patients with Child-Pugh A and ECOG 0–1), the combination of cadonilimab and lenvatinib demonstrated an ORR of approximately 35.5–35.7% and a DCR of around 90–93%. Although the comparison with lenvatinib monotherapy (mRECIST ORR approximately 24%) or atezolizumab + bevacizumab (RECIST 1.1 ORR approximately 27%) is indirect and exploratory, the combination still shows promising antitumor activity (27, 37, 38).Grade ≥3 TRAEs were frequent (e.g., hypertension, proteinuria, transaminase elevations) in keeping with lenvatinib-based regimens, underscoring the need to balance exposure and toxicity in future trials (27). In another small-sample 1b/II cohort (n=24), the ORR was 16.7% (all partial responses, no complete responses). While this study has certain limitations, cadonilimab still demonstrates some efficacy in the treatment of HCC (20).

At present, although preclinical and clinical studies on cadonilimab for the treatment of HCC remain limited, existing evidence preliminarily suggests that it possesses therapeutic potential in advanced HCC, offering a new avenue for clinical application. Several clinical trials evaluating cadonilimab in HCC are currently underway in China (ChiCTR2300077787, ChiCTR2300068781, ChiCTR2200067161, ChiCTR2400080364), which are expected to provide valuable evidence to support its use in the treatment of HCC.

### 2.3 Cadonilimab in lung cancer: research progress and clinical application

Case reports have indicated that cadonilimab demonstrates promising efficacy in patients with advanced lung cancer who developed resistance to prior immunotherapy (39). In addition, cadonilimab combined with anlotinib has shown preliminary activity in a single-arm phase Ib/II study (NCT04646330) of cadonilimab plus anlotinib for PD-L1 TPS ≥1% NSCLC (n=18), with ORR 62.5%, DCR 100%, and grade ≥3 TRAEs 6%; sample size and follow-up are limited, so findings are hypothesis-generating. Among the five patients with non-squamous NSCLC, the ORR reached 80%, while the incidence of grade 3 TRAEs was only 6%. This Ib/II trial (NCT04646330) is a small-sample, single-arm study. Although it provides preliminary signals rather than confirmatory evidence, the study has shown positive results (40).

Subsequently, the study NCT04172454 evaluated the efficacy and safety of cadonilimab in previously treated NSCLC patients. Participants were assigned to three cohorts: cohort A included patients who had failed platinum-based doublet chemotherapy but had not received prior immuno-oncology (IO) therapy; cohort B included patients who failed platinum-based doublet chemotherapy and exhibited primary resistance to IO; and cohort C included patients who failed platinum-based doublet chemotherapy and developed acquired resistance to IO. The ORR in cohort A was 10%, while no CR or PR were observed in cohorts B and C. The median overall survival (OS) was 19.61 months in cohort A, 4.93 months in cohort B, and 13.16 months in cohort C. The incidence of grade ≥3 TRAEs was 11.3%, most commonly involving liver enzyme abnormalities and hematologic toxicities (41). Although no responses were observed in patients with primary or acquired resistance to IO, cadonilimab demonstrated potential antitumor activity in previously treated NSCLC patients (42). These findings provide further support for the clinical development of cadonilimab in NSCLC.

Recent findings suggest that cadonilimab may offer therapeutic potential as a subsequent-line option for patients with advanced lung adenocarcinoma who develop resistance to IO therapy. In one reported case, a patient with advanced lung adenocarcinoma received cadonilimab in combination with chemotherapy after IO resistance. Following four cycles of treatment, the patient achieved a PR, with stable disease and no progression, reaching a progression-free survival (PFS) of 8.1 months, along with a marked improvement in quality of life (39). Ongoing clinical trials in China are further investigating the use of cadonilimab in lung cancer, which may provide new therapeutic options for patients in the future.

### 2.4 Cadonilimab in gastric cancer: research progress and clinical application

In clinical studies of cadonilimab combined with chemotherapy (oxaliplatin and capecitabine) for advanced gastric/gastroesophageal junction cancer (G/GEJ), encouraging results have been achieved, representing a milestone in the immunotherapy of gastric cancer. The study reported anORR of 66.7% and a DCR of 95.8%, with an overall incidence of TRAEs of 79.4% and grade 3 TRAEs occurring in 29.4% of patients, predominantly characterized by reductions in blood cell counts Subsequently, Ji et al. also observed in advanced G/GEJ patients treated with cadonilimab plus chemotherapy an ORR of 68.2%, a CRR of 5.7%, a PRR of 62.5%, and a DCR of 92.0%. In this study, grade ≥3 TRAEs occurred in 69.4% of patients, mainly involving myelosuppression and gastrointestinal toxicitie (43). These findings suggest that cadonilimab in combination with chemotherapy has the potential to become a new first-line therapeutic option for advanced G/GEJ cancer. A 2025 MSI-H gastric cancer salvage case using cadonilimab plus apatinib further highlights activity beyond standard settings (44).

A male patient with stage IV gastroesophageal junction (GEJ) cancer was enrolled in a clinical trial of cadonilimab combined with chemotherapy, which required participants to have an unknown or negative human epidermal growth factor receptor 2 (HER2) status. This patient, however, received treatment prior to undergoing HER2 testing. Subsequent evaluation revealed HER2 positivity, and remarkably, the patient achieved a CR following cadonilimab plus chemotherapy. Although treatment was later discontinued, disease assessment continued to show a PR (45). This unique case highlights cadonilimab as a novel and effective therapeutic option for advanced HER2-positive GEJ cancer, warranting consideration as a potential treatment strategy. The 2024 Nat Med phase 1b/2 COMPASSION-04 trial in HER2-negative G/GEJ adenocarcinoma reported ORR 68.2% and DCR 92.0% for cadonilimab plus chemotherapy (43). Together, these findings suggest that cadonilimab plus chemotherapy demonstrates promising efficacy and manageable safety in the treatment of G/GEJ cancer.

These findings provide strong evidence to support ongoing research in gastric cancer and suggest that cadonilimab may become the first bispecific antibody worldwide to usher in a new era of immunotherapy for gastric cancer.

### 2.5 Cadonilimab in other cancers: research progress and clinical application

In recent years, research on cadonilimab in other malignancies has expanded, yielding breakthrough progress. The NCT03261011 study aimed to evaluate the efficacy of cadonilimab in patients with advanced solid tumors. Results showed an overall ORR of 13.4% across all enrolled patients, with two cases achieving CR. Among the advanced solid tumor cohort, 16.8% (20 cases) were mesothelioma, with an ORR of 20% and a DCR of 75.0%. In addition, the most common adverse event was infusion-related reaction (18.5%); immune-related AEs occurred in 44.5% (any-grade), and grade ≥3 irAEs were 6.7% (46).

A phase II clinical trial (NCT04220307) evaluated cadonilimab in patients with previously treated recurrent or metastatic nasopharyngeal carcinoma (R/M-NPC). Among the 23 enrolled patients, efficacy assessment showed an ORR of 26.1% and a DCR of 56.5%. Subgroup analysis revealed that patients with PD-L1 TPS ≥50% achieved an ORR of 44.4%, whereas those with PD-L1 TPS <50% had an ORR of 14.3%. The incidence of grade ≥3 TRAEs was 8.7%, with the most common adverse events being hypothyroidism and rash (47). These findings indicate that cadonilimab monotherapy demonstrates encouraging efficacy in R/M-NPC and offers a novel therapeutic option for this patient population.

## 3 Problems and challenge

Resistance considerations: in AK104-202, primary/acquired IO-resistant NSCLC cohorts (B/C) showed no ORR, whereas cadonilimab plus anlotinib achieved ORR 62.5% with DCR 100% and real-world pretreated cohorts also reported activity, suggesting some resistance can be overcome via vascular/TME modulation. Likely mechanisms include low PD-1/CTLA-4 density, T-cell exclusion or antigen-presentation loss, and immunosuppressive myeloid/Treg infiltration. Prospective trials should enroll IO-resistant populations, test TME-remodeling combinations (anti-angiogenic, chemotherapy/radiation), and embed serial tumor/blood profiling to map and intercept escape pathways.

Key gaps remain: evidence is dominated by phase I/II single-arm data with few randomized comparisons and limited follow-up (**Table 1**); actionable biomarkers beyond PD-L1 are sparse despite signals in HER2-positive GEJ cancer and MSI-H gastric cancer; optimal dosing, sequencing, and perioperative use are undefined; long-term irAE risk and management strategies require stronger protocols; and real-world effectiveness in PD-1/PD-L1–resistant disease is uncertain. Suggested approaches include launching adequately powered randomized trials (including IO-resistant cohorts) and perioperative studies to test pathologic response and DFS; incorporating biomarker programs (HER2, MSI-H, PD-L1 TPS, TME/immune signatures) into trial designs; evaluating step-up or weight-based dosing to balance efficacy and irAEs; and establishing standardized monitoring/mitigation pathways for immune toxicities, informed by emerging real-world data.

**Table 1:** Clinical trials of cadonilimab monotherapy or combination therapy for malignant tumors.

Study/Trial	Indication	Line/Design	Regimen	ORR %	DCR %	Grade≥3TRAEs%
COMPASSION-13	R/M cervical cancer	Phase Ib/II single-arm	Cadonilimab + chemo ± bev 10–15 mg/kg Q3W	66.7–92.3	—	~20–27
Yu 2024 case	Metastatic cervical cancer	Post-chemo intolerance	Cadonilimab mono	CR (case)	—	—
COMPASSION-08	Advanced HCC	Phase Ib/II single-arm	Cadonilimab + lenvatinib 6 mg/kg Q2W or 15 mg/kg Q3W	35.5–35.7	90–93	Frequent ≥3 AEs (HTN/proteinuria/LFT↑)
AK104-202	NSCLC post-platinum	Phase Ib/II single-arm	Cadonilimab mono	10% (A); 0% (B/C)	—	11.3
NCT04646330	NSCLC PD-L1 TPS ≥1%	Phase Ib/II single-arm	Cadonilimab + anlotinib	62.5	100	6
COMPASSION-04	HER2–G/GEJ adenocarcinoma	Phase Ib/II single-arm	Cadonilimab + chemo	68.2	92	High (mainly myelosuppression/GI)
COMPASSION-01	Advanced solid tumors	Phase 1a/1b	Cadonilimab mono	13.4 overall; mesothelioma 20	75 (meso)	≥3 irAEs 6.7 (any-grade irAEs 44.5)
COMPASSION-06	R/M nasopharyngeal carcinoma	Phase II	Cadonilimab mono	26.1	56.5	8.7

## 4 Conclusions and future perspectives

Although cadonilimab, a bispecific antibody targeting PD-1 and CTLA-4, shows promising early antitumor activity, it is still largely supported by phase I/II evidence. Future work should clarify cadonilimab’s role in PD-1/PD-L1 inhibitor–resistant disease (limited ORR in primary/acquired IO-resistant NSCLC but signals with anlotinib and real-world pretreated cases), test perioperative use in neoadjuvant/curative-intent settings beyond metastatic cohorts, and prioritize biomarkers beyond PD-L1—including signals in HER2-positive GEJ, MSI-H gastric cancer, and PD-L1 TPS–stratified NSCLC—to refine patient selection. By simultaneously blocking two immune checkpoints, it synergistically enhances T-cell activation and antitumor responses, leading to meaningful clinical benefits in multiple solid tumors, including cervical cancer, gastric cancer, hepatocellular carcinoma, and non-small cell lung cancer. Across studies, subgroup signals in PD-L1–negative tumors are exploratory and based on small cohorts (e.g., cervical cancer ORR 16.7% vs 43.8% in PD-L1–positive; variable PD-L1 TPS strata in NSCLC), so any PD-L1–independent activity remains unconfirmed and needs larger, powered analyses. Early studies also highlight the potential role of biomarkers such as HER2 in guiding individualized treatment. Compared to other PD-1/CTLA-4 bispecific antibodies discussed in this article, the tumor-selective binding of Cadonilimab may result in lower systemic immune-related adverse events. With its favorable safety profile and sustained efficacy, it holds significant clinical value.

However, current evidence is largely derived from phase II trials with limited sample sizes, and high-level evidence from large, multicenter, randomized phase III trials remains lacking. The mechanisms of primary and acquired resistance are not yet fully understood, and optimal strategies for overcoming resistance require further study. In addition, the best approaches for integrating cadonilimab into combination regimens, including with chemotherapy, targeted therapy, radiotherapy, and other immunotherapies, remain to be defined. Dose optimization, sequencing, and long-term safety management are also unresolved questions.

Looking forward, future research should focus on rational combination strategies informed by mechanistic insights, as well as the development of predictive models based on integrated biomarker and multi-omics analysis to enable more precise patient selection. Expanding the scope of cadonilimab to rare tumors and special populations, alongside real-world data collection, will be critical to validating its therapeutic value. Moreover, the establishment of standardized protocols for monitoring and managing rare immune-related adverse events will help ensure patient safety.

Despite existing challenges, the accumulating clinical and translational evidence supports cadonilimab as a promising therapy with the potential to broaden indications, refine treatment strategies, and improve long-term survival outcomes for patients with malignancies.
